# Non-Mammalian Models for Mitochondria Research in CNS Disorders

**DOI:** 10.3390/biom16071072

**Published:** 2026-07-22

**Authors:** Dubravka Svob Strac, Vedrana Filic, Ana Filosevic Vujnovic, Ivana Vrhovac Madunic, Josip Madunic, Ana Cipak Gasparovic, Ana Havelka Mestrovic, Rozi Andretic Waldowski

**Affiliations:** 1 Ruđer Bošković Institute, 10000 Zagreb, Croatia; vedrana.filic@irb.hr (V.F.); acipak@irb.hr (A.C.G.); 2 Faculty of Biotechnology and Drug Development, 51000 Rijeka, Croatia; ana.filosevic@uniri.hr (A.F.V.); randretic@uniri.hr (R.A.W.); 3 Division of Toxicology, Institute for Medical Research and Occupational Health, 10000 Zagreb, Croatia; ivrhovac@imi.hr (I.V.M.); jmadunic@imi.hr (J.M.); 4 Department of Psychology, Algebra Bernays University, 10000 Zagreb, Croatia; ana.havelkamestrovic@algebra.hr

**Keywords:** CNS disorders, mitochondria, non-mammalian models, *Saccharomyces cerevisiae*, *Dictyostelium discoideum*, *Caenorhabditis elegans*, *Drosophila melanogaster*, *Danio rerio*

## Abstract

Mitochondrial dysfunction is increasingly recognized as a major contributor to central nervous system (CNS) disorders, including neurodegenerative and neuropsychiatric diseases. Animal models are essential for elucidating disease mechanisms and supporting the development of new therapeutic strategies. Among these models, non-mammalian organisms offer distinct advantages, including low cost, rapid life cycles, genetic tractability, and suitability for large-scale, high-throughput studies. Organisms such as *Saccharomyces cerevisiae*, *Dictyostelium discoideum*, *Caenorhabditis elegans*, *Drosophila melanogaster*, and *Danio rerio* have substantially advanced the understanding of mitochondrial processes relevant to CNS pathology. Studies using these models have revealed conserved mechanisms involving mitophagy, mitochondrial quality control, respiratory function, bioenergetic signaling, and neurodegenerative pathways. Their strengths, including scalability, live imaging capacity, and efficient genetic manipulation, have accelerated disease modeling and therapeutic discovery. However, simplified physiology, evolutionary distance from humans, and the incomplete representation of complex CNS organization limit their translational relevance and often require validation in higher-order organisms. Nevertheless, integrating these models into CNS research, particularly alongside emerging technologies, provides a powerful strategy for linking fundamental mitochondrial biology with translational neuroscience. This review summarizes the use of non-mammalian models in neuroscience research, with an emphasis on mitochondrial dysfunction in CNS disorders and their potential to support future therapeutic advances.

## 1. Mitochondrial Dysfunction in CNS Disorders

The central nervous system (CNS) has exceptionally high metabolic demands, making neurons highly dependent on proper mitochondrial integrity and function. Mitochondria are essential for neuronal survival and function because they regulate ATP production required for synaptic transmission, calcium homeostasis, redox balance, lipid metabolism, apoptotic signaling, and overall neuronal viability [1]. Mitochondrial quality control is also crucial for preserving cellular homeostasis. When mitochondrial membrane potential collapses, damaged organelles are selectively removed through mitophagy, a process required to maintain mitochondrial integrity and cellular function. Disruption of this pathway has been implicated in a wide range of pathological conditions [2]. Given these pivotal roles, mitochondrial dysfunction is increasingly implicated in the pathophysiology of many CNS disorders, including both neurodegenerative and neuropsychiatric diseases [3]. Growing evidence indicates that mitochondrial dysfunction is not only a downstream consequence of disease but may also act as a primary driver of pathogenesis, by contributing to neuronal degeneration, synaptic dysfunction, and neuroinflammation. To date, more than 150–350 distinct diseases have been associated with mitochondrial dysfunction, including a wide range of CNS disorders such as Alzheimer’s disease (AD), Huntington’s disease (HD), Parkinson’s disease (PD), amyotrophic lateral sclerosis (ALS), Leigh syndrome (LS), NARP (neuropathy, ataxia, and retinitis pigmentosa) syndrome, and hereditary spastic paraplegia [4,5]. Primary mitochondrial diseases constitute a heterogeneous group of genetic disorders characterized by impaired mitochondrial function resulting from mutations in either mitochondrial DNA (mtDNA) or nuclear DNA (nDNA), which encode mitochondrial structural or functional components. These mutations disrupt key mitochondrial processes, including oxidative phosphorylation (OXPHOS), mitochondrial dynamics, and protein import. A substantial proportion of genes implicated in neurodegenerative disorders are directly linked to mitochondrial pathways, underscoring the critical importance of mitochondrial integrity for neuronal survival and function [6,7,8]. In addition to primary mitochondrial disorders, many genetic and non-genetic diseases, including numerous CNS conditions, exhibit mitochondrial dysfunction as a secondary pathological feature. In these cases, mitochondrial impairment may arise from processes such as protein aggregation, intracellular calcium overload, and oxidative stress. CNS disorders are typically multifactorial, and mitochondrial dysfunction often represents a convergent mechanism that exacerbates disease progression [6,7,9]. This dysfunction is commonly characterized by impaired respiratory chain activity, excessive production of reactive oxygen species (ROS), altered mitochondrial dynamics, and defective quality control mechanisms, such as mitophagy. These alterations contribute to neuronal damage and degeneration, even when mitochondrial dysfunction is not the primary etiological factor [6,7,9].

## 2. Animal Models in CNS Research

Neuroscience research relies extensively on animal models to advance our understanding of neural function, behavior, and the molecular mechanisms underlying neuropsychiatric and neurodegenerative disorders. These models are critical for elucidating CNS disease pathogenesis and for supporting the development and preclinical evaluation of new therapeutic strategies. The selection of appropriate animal models is guided by practical and scientific considerations, including experimental throughput, reproductive capacity, developmental timeline, ease of maintenance, availability of genomic and transcriptomic resources, susceptibility to genetic manipulation, and similarity to the human nervous system [10]. Although rodents, particularly mice and rats, remain the most widely used models in neuroscience, comparative studies across species are essential for distinguishing conserved biological mechanisms from species-specific features [11]. Non-mammalian model organisms provide simplified systems for elucidating fundamental neurobiological processes. These models are especially valuable for studying complex mechanisms associated with neuropsychiatric disorders, which are often difficult, time-consuming, and costly to examine in mammalian systems. Importantly, non-mammalian organisms enable high-throughput hypothesis testing and are frequently used in early-stage investigations of disease pathways, biomarker discovery, and drug screening before validation in higher-order models [10,12]. Moreover, non-mammalian animal models align closely with ethical frameworks and the principles of the 3Rs in animal experimentation because they offer valuable alternatives to traditional mammalian models while reducing ethical concerns associated with the use of higher vertebrates [13]. They can substitute for mammalian species in early-stage mechanistic and neurodevelopmental studies (replacement), and their high reproductive output supports statistically robust experiments with fewer breeding animals and lower overall animal use (reduction). Refinement is supported through their simpler husbandry requirements, lower perceived capacity for suffering, and suitability for non-invasive imaging and high-throughput analyses. Zebrafish embryos, for example, are frequently not considered protected experimental animals before reaching independent feeding stages, supporting their ethical utility.

## 3. Non-Mammalian Models to Study Mitochondrial Dysfunction in CNS

A key advantage of non-mammalian models for studying mitochondrial dysfunction in CNS disorders is the strong evolutionary conservation of mitochondrial genes and pathways between these organisms and humans. This conservation enables investigation of essential processes such as OXPHOS, mitochondrial dynamics, mitophagy, mitochondrial unfolded protein response (UPRmt), and redox signaling, thereby providing translational insights into human disease [14]. Species such as *Saccharomyces cerevisiae*, *Dictyostelium discoideum*, *Caenorhabditis elegans*, *Drosophila melanogaster*, and *Danio rerio* have proven invaluable in elucidating the molecular and cellular mechanisms underlying mitochondrial dysfunction in CNS diseases. The following sections provide an overview of these non-mammalian models and their applications in neuroscience research, with a particular emphasis on mitochondrial dysfunction in CNS disorders.

### 3.1. Saccharomyces cerevisiae

*Saccharomyces cerevisiae*, a single-cell eukaryote, is widely used in molecular biology and biochemistry. Yeast shares several practical advantages with bacteria, including rapid division, a well-characterized and relatively simple genome (approximately 6000 genes), straightforward genetic manipulation and suitability for large-scale experiments [15]. At the same time, as a eukaryote, yeast provides an excellent system for studying post-translational modifications and protein folding. In addition, deletion-mutant collections containing more than 4800 gene deletions are available [16] and nearly half of the essential yeast genes can be replaced by human homologs [17]. The yeast mitochondrial genome is a circular DNA ranging from approximately 78,000 bp in shorter genomes to 85,000 bp in longer genomes, mainly because of strain-specific variation in intron and non-coding-region length [18]. Yeast mtDNA can also undergo massive deletions (ρ^-^ mutants), or complete loss (ρ^0^ mutants), resulting in “petite” phenotype characterized by tiny colonies on agar plates [19]. This combination of features makes yeast a valuable model for studying the metabolic and signaling pathways involved in eukaryotic diseases while retaining many advantages of bacterial systems, such as high-throughput capacity, genetic tractability, and experimental efficiency. Yeast cultivation is also substantially less expensive than that of mammalian cell culture, providing a cost-effective yet powerful system for investigating fundamental cellular processes. However, yeast has important limitations, particularly for studies of cell–cell interactions, tissue organization, or organ-related physiology, which cannot be replicated in this unicellular system.

The advantages of yeast as a model organism in biological and biochemical research have driven the development of yeast-based systems for studying neurodegenerative disorders. Its ease of genetic manipulation, short life cycle, conservation of fundamental eukaryotic cellular pathways, and the presence of endogenous amyloid and prions [20], enabled the establishment of the first yeast model of HD [21]. Since then, several HD yeast models have been developed and successfully used as high-throughput platforms for screening potential therapeutic compounds [22,23]. In addition, a yeast model expressing huntingtin fragments containing flanking polyglutamine (polyQ) regions has been established to investigate huntingtin protein aggregation, providing insights into the mechanisms governing the transition between benign and toxic protein conformations [24]. ALS is another neurodegenerative disorder for which several yeast models have been developed [25]. Although ALS pathology is complex, most patients exhibit defects in fundamental cellular processes, including dysregulated RNA metabolism and protein folding, making yeast an effective system for investigating these pathways and the functional consequences of disease-associated mutations [25]. However, because yeast lacks an immune system, inflammation mechanisms contributing to ALS pathology cannot be modeled in this system. Similarly, yeast models have been developed for PD, which is characterized by the intracellular aggregation of α-synuclein, despite the absence of a yeast ortholog of α-synuclein. These models are generated by expressing α-synuclein fused to the green fluorescent protein (GFP) under the control of an inducible promoter, allowing protein expression to be switched on or off by changing the growth medium from glucose to galactose [26]. Considering the evolutionary origin of mitochondria, it is not surprising that yeast and humans share approximately 70% of nuclear-encoded mitochondrial genes, many of which are implicated in mitochondrial disorders [27]. Consequently, essential mitochondrial processes including mitochondrial import, mitochondrial dynamics, quality control, and mitochondria-derived signaling are highly conserved across these species [28]. One of the key advantages of yeast is its ability to survive without functional OXPHOS due to its capacity for fermentation [29,30], enabling the analysis of otherwise lethal mutations and deletions in essential mitochondrial genes [31].

In PD, for example, two mitochondrial Hsp70 variants, P509S and R126W, have been shown to contribute to disease susceptibility [32]. In yeast models, these variants exhibited increased aggregation resulting from reduced protein stability or enhanced interactions with J-proteins, thereby recapitulating the cellular defects observed in PD patients [32]. Yeast models have also provided valuable insights into the molecular mechanisms underlying ALS. The first gene linked to ALS was *SOD1*, which encodes copper/zinc superoxide dismutase. Although more than 200 *SOD1* mutations have been identified in ALS patients, yeast strains expressing human *SOD1* in place of the endogenous yeast ortholog demonstrated that all these mutant variants remain functional [33]. Furthermore, these models revealed that ALS-associated *SOD1* mutants preferentially aggregate near mitochondria due to factors such as reduced acetylation of Lys 123 or modifications of His47, His49, His64, His72, His81, and His121, which compromise protein stability and conformation, thereby promoting aggregation [34]. In studies of LS, yeast has served as a valuable model for validating mitochondrial defects associated with the disease. For example, yeast-based assays confirmed the pathogenicity of two novel *OPA1* mutations identified in patients with early-onset LS [35], as well as a splice variant in the *ATP5PO* gene associated with the syndrome [36]. Interestingly, certain point mutations are shared among multiple CNS-related mitochondrial diseases. One notable example is *MT-ATP6* gene (yeast ortholog: *ATP6*), in which mutations have been associated with ataxia, NARP and maternally inherited LS (MILS) [37,38]. *MT-ATP6* point mutations severely impair ATP synthase function, and yeast models have demonstrated that these defects also reduce the levels of Complex IV, closely recapitulating the mitochondrial disfunction observed in affected patients [37,39,40]. In addition to neurodegenerative diseases, yeast is an exceptionally valuable system for studying primary mitochondrial disorders, particularly mitochondrial maintenance disorders such as progressive external ophthalmoplegia (PEO). The molecular basis of PEO is linked to mutations in the DNA polymerase γ catalytic subunit encoded by *POLG* [41]. The yeast ortholog *Mip1*, together with the ability of yeast to survive the complete loss of mtDNA through petite colony formation, provides an exceptionally attractive system for investigating the functional consequences of DNA polymerase γ mutations [41]. Overall, *S. cerevisiae* is an affordable and powerful model for investigating the molecular mechanisms of CNS mitochondria-related disorders due to its easy genetic manipulation, evolutionary conserved pathways, the ability to tolerate mitochondrial dysfunction, suitability for studying pathogenic processes, identifying therapeutic targets and high-throughput screening for candidate therapeutics (Table 1).

### 3.2. Dictyostelium discoideum

Another simple yet valuable model in mitochondrial research related to human CNS disorders is the protist *Dictyostelium discoideum*. This amoeba naturally lives in soil, feeds by phagocytosing bacteria, and grows through mitotic division. Laboratory strains possess mutations that strongly upregulate fluid uptake, enabling axenic growth in liquid culture medium. *D. discoideum* is remarkable because, under unfavorable conditions, it transitions from isolated vegetative cells to multicellular development and produces spores. Its nuclear [42] and mitochondrial [43] genomes have been fully sequenced. Vegetative *D. discoideum* cells are haploid and contain six chromosomes encoding approximately 12,500 genes [42]. Molecular tools for gene editing by homologous recombination [44] or CRISPR/Cas [45] are readily available, and protocols for standard molecular biology techniques, cellular assays, and cell imaging are well established. Together with its unicellular and multicellular lifestyles, these features make *D. discoideum* an attractive model for studying cell motility, endocytosis, cytokinesis, adhesion, multicellularity, social behavior, and human diseases [46]. *D. discoideum* emerged from the animal-fungal lineage after the split between plants and animals but before the fungal lineages diverged from animals [42,47]. Nevertheless, many of its proteins are more similar to human proteins than those from *S. cerevisiae*, and it contains numerous genes orthologous to human disease-related genes [42,48]. This has led the National Institutes of Health (NIH) in the United States to recognize *Dictyostelium* as an important non-mammalian biomedical model [49]. As a model for human pathology, *D. discoideum* has been used to study bacterial infections [50], amoeboid migration relevant to immune and cancer cells [51,52], extracellular vesicles in intercellular communication, drug delivery and detoxification [53], endoplasmic reticulum (ER) stress responses [54], autophagy [55], cancer [56], mitochondrial disorders [57], and neurological diseases [58].

The *D. discoideum* mitochondrial genome is a 55.6 kb circular DNA molecule comprising 61 genes, making it substantially larger than the highly reduced human mitochondrial genome, which is approximately 16.6 kb and contains 37 genes [43,59]. All genes are located on the same DNA strand and share a common transcriptional orientation, primarily encoding proteins and RNAs required for respiration and translation. To study mitochondrial disorders in *D. discoideum*, several approaches have been developed to induce sublethal inhibition of the expression of nuclear genes encoding mitochondrial proteins, or mitochondrial genes encoding proteins required for mitochondrial protein translation, respiratory chain function, and OXPHOS. These approaches include the knockout of nuclear genes encoding non-essential mitochondrial proteins, RNA interference or antisense inhibition of mitochondrial proteins, and ethidium bromide treatment to deplete cells of mtDNA [60]. Because each *D. discoideum* cell contains approximately 200 copies of the mitochondrial genome, a strategy has also been developed to mimic heteroplasmy observed in human mitochondrial diseases [61]. This approach relies on homologous recombination between mtDNA and a plasmid vector carrying a 500–1000 bp fragment of the targeted gene. However, a limitation of this method is the potential for off-target incorporation into the nuclear genome, which must be considered when interpreting the transformant phenotypes. Whereas the relationship between genetic mutations and phenotype is often complex and poorly understood in human mitochondrial diseases, *D. discoideum*, provides a genetically tractable system for reproducible phenotypic analysis of mitochondrial disease states because it has a haploid genome and displays numerous phenotypes throughout its short life cycle. In addition, a method for measuring mitochondrial respiratory function in *D. discoideum* has been established [62]. Phenotypes associated with OXPHOS-related mitochondrial dysfunction in *D. discoideum* include impaired growth in liquid culture medium and on bacterial lawns without effects on macropinocytosis or phagocytosis; impaired cell aggregation during the transition to multicellular development; defective phototaxis and thermotaxis during the multicellular slug stage; and disturbed cell differentiation during multicellular development, with an increased number of stalk cells [60]. These phenotypes have been observed in most mitochondrially diseased cells, regardless of the underlying mutation [63,64,65]. Based on phototaxis- and thermotaxis-deficient mitochondrial mutants, *Dictyostelium* researchers were the first to propose that defective mitochondria affect cellular signaling [63]. It was later shown that the phenotypes of mitochondrially diseased *D. discoideum* cells are induced by constitutively active AMP-activated protein kinase (AMPK), a sensor of cellular energy levels, rather than by decreased ATP levels or reduced mitochondrial mass [66,67,68]. Chronic AMPK activation has also been demonstrated in neurons of patients with PD, AD, and HD [69].

*D. discoideum* has been used as a model for both primary mitochondrial diseases and diseases of non-mitochondrial origin that affect mitochondrial function [57]. For example, *D. discoideum* has been utilized as the first NDUFAF5 model to study LS, since it contains a highly similar protein, crucial for the function of complex I of the electron transport chain [70,71,72,73,74]. The *ndufaf5* null cells showed a significant reduction in complex I respiratory activity, a marked growth defect, defective slug phototaxis and delayed development, resulting in non-viable spores. In contrast to other mitochondrially diseased cells, the phenotypes of *ndufaf5* null cells are not caused by increased AMPK activity. Nevertheless, bulk autophagy was increased, representing the first evidence linking autophagy to complex I defects [74]. An example of a non-mitochondrial disease that affects mitochondrial function and has been investigated using *D. discoideum* is PD, with 5–10% of cases being hereditary due to mutations in *PARK* genes [75,76]. Mutations in *DJ-1* (*PARK7*), *LRRK2* (*PARK8*), and *HTRA2* (*PARK13*) have been modeled in *D. discoideum* [77,78,79] (Table 2). Although loss-of-function mutations in *DJ-1* cause mitochondrial dysfunction, a major contributor to PD pathogenesis [76,80], studies in *D. discoideum*, which contains a single *DJ-1* homolog encoded by the *deeJ* gene, have demonstrated a non-mitochondrial role for DJ-1 in unstressed cells as a positive regulator of phagocytosis and growth on bacterial lawns [81]. Interestingly, *DJ-1* knockdown does not impair mitochondrial function but instead increases mitochondrial respiration, and the overall phenotype does not mimic typical mitochondrial dysfunction [81]. Under oxidative stress, however, these cells exhibit AMPK-dependent phenotypes characteristic of mitochondrial disease, indicating that DJ-1 protects cells from AMPK hyperactivation during oxidative stress [77]. Alternatively, *D. discoideum* can be used to investigate the functions of human disease-related proteins that lack homologs in this organism. For example, the cytotoxicity of human neurodegeneration-associated proteins tau and α-synuclein has been examined in naïve *D. discoideum* cells [82]. Additional examples of mitochondrial dysfunctions associated with neurological disorders studied in *D. discoideum* are also listed in Table 2.

### 3.3. Caenorhabditis elegans

The nematode *Caenorhabditis elegans* (*C. elegans*) is a small, transparent metazoan that has emerged as a fundamental model for neurobiology and disease research [89]. It was the first organism to have its entire synaptic connectome reconstructed [90,91], enabling single-cell and synapse-level analysis of neural circuits. Despite this simplicity, its nervous system exhibits architectural features such as small-world topology, modular organization, and hub “rich-club” connectivity, a network property in which highly connected hub neurons are preferentially interconnected and act as central communication nodes linking distinct neuronal modules. In addition, *C. elegans* possesses an extensive “wireless” signaling architecture based on neuropeptide–receptor interactions that resembles signaling systems found in mammalian brains [92,93,94,95]. The organism’s transparency facilitates real-time imaging of mitochondrial dynamics, synaptic transmission, and protein aggregation within an intact multicellular context [96,97,98,99,100]. *C. elegans* genome is fully sequenced, and approximately 40–80% of human genes, including 40–50% of disease-associated genes, have orthologs in *C. elegans*. Many of these genes are involved in lipid metabolism and insulin/IGF-1, AMPK, and mTOR signaling [101,102,103,104]. This high degree of conservation, together with advanced molecular tools including RNA interference, CRISPR/Cas editing, and transgenics [105,106,107], enables high-throughput genetic and pharmacological screening. The genome encodes more than 150 neuropeptide precursors and a similar number of peptide-activated G protein-coupled receptors [108,109], reflecting remarkable biochemical diversity and molecular conservation with humans. The nematode develops rapidly, progressing from embryo to adult in only three days. Combined with the complexity of a multicellular organism, this feature makes it ideal for studying cellular differentiation, development, apoptosis, autophagy, and mitochondrial quality control [110,111,112]. Its short life cycle, small body size, and low maintenance cost make *C. elegans* a versatile and powerful system for investigating aging, metabolism, stress responses, reproductive biology, host–microbiome interactions, and toxicology [113,114,115,116,117,118,119,120]. Behavioral outputs such as locomotion, chemotaxis, learning, and mechanosensation provide robust functional readouts of neuronal integrity and connectivity, making *C. elegans* an indispensable model for linking molecular perturbations to organismal phenotypes associated with CNS disorders [121,122]. Transgenic strains expressing human neurodegeneration-associated proteins, including α-synuclein (PD) [123,124], amyloid-β (Aβ) and tau (AD), polyglutamine (HD), and mutant SOD1 or TDP-43 (ALS) [125], have been used to identify genetic and pharmacological factors that alleviate neuronal loss, many of which exert their effects on mitochondrial homeostasis, autophagy, and proteostatic regulation [126,127].

*C. elegans* also provides a uniquely powerful in vivo system for investigating mitochondrial biology [128]. Mitochondrial dynamics in *C. elegans* are tightly regulated by *FZO-1*, *EAT-3* and *DRP-1*, the orthologs of mammalian mitofusins (*MFN1/2*), *OPA1*, and human *DNM1L/Drp1*, respectively [129]. Disruption of these genes results in fragmented or hyperfused mitochondrial networks, impaired ATP production, and neurobehavioral deficits, highlighting their critical roles in maintaining neuronal homeostasis [130]. Disturbances in mitochondrial dynamics often signal selective organelle degradation through mitophagy, which is regulated by the kinase PINK-1 and the ubiquitin ligase PDR-1, functional orthologs of human PINK1 and Parkin [131]. Loss of PINK-1 or PDR-1 causes accumulation of depolarized mitochondria, elevated oxidative stress, and dopaminergic neurodegeneration, closely resembling PD pathology [132]. Another conserved pathway investigated in *C. elegans* is UPRmt, a stress-response pathway that “detects” mitochondrial dysfunction and activates the nuclear transcriptional factor ATFS-1, leading to the upregulation of chaperones HSP-6 and HSP-60, to restore mitochondrial function [133,134]. *C. elegans* has also been widely used to investigate surveillance systems that integrate mitochondrial function with cellular metabolism and longevity pathways, including AMPK, sirtuins, and insulin/IGF-1 signaling (IIS) [135,136,137]. Moreover, *C. elegans* is highly sensitive to mitochondrial toxins, including rotenone, paraquat, and 1-methyl-4-phenylpyridinium (MPP^+^), making it a well-established model for exploring how environmental and chemical insults cause mitochondrial stress and neuronal damage [138]. Table 3 summarizes representative *C. elegans* models of CNS diseases and primary mitochondrial disorders, highlighting the corresponding genetic or transgenic manipulations and the key phenotypes observed in each model.

In *C. elegans* models of PD, expression of human α-synuclein in dopaminergic neurons or mutations in the *PINK-1* and *Parkin* orthologs (*pdr-1*) cause progressive dopaminergic neurodegeneration accompanied by mitochondrial fragmentation, impaired respiration, and excessive ROS production [112,139], mirroring mitochondrial pathology in human PD. In AD models, neuronal expression of human Aβ or tau leads to mitochondrial fragmentation, reduced ATP synthesis, increased oxidative stress, and disrupted calcium homeostasis [140], contributing to synaptic failure and neuronal dysfunction and resembling early AD pathology. Activation of UPRmt and upregulation of mitochondrial chaperones such as HSP-6 and HSP-60 mitigate these phenotypes by restoring energy metabolism and reducing oxidative damage [141,142]. In *C. elegans* models of HD, polyQ expanded fragments of the human huntingtin protein cause mitochondrial dysfunction characterized by an altered fission–fusion balance, impaired respiratory capacity, and elevated ROS levels in GABAergic and cholinergic neurons [143,144,145]. These defects recapitulate HD-related mitochondrial pathology and correlate with behavioral and locomotor impairments. In ALS, *C. elegans* models expressing mutant *SOD1*, *TDP-43*, or *FUS* demonstrate impaired mitochondrial transport along axons, accumulation of oxidatively damaged mitochondria, and defective mitophagy, resulting in motor neuron degeneration [125,146]. Together, these models closely parallel mitochondrial dysfunction observed in human neurodegenerative disorders and enable high-throughput screening of compounds with neuroprotective potential. Beyond well-studied neurodegenerative diseases, *C. elegans* has also been widely used to model primary mitochondrial diseases affecting the CNS. Nematode homologs of genes implicated in human LS and Friedreich’s ataxia (FRDA) recapitulate key pathological features, including impaired electron transport chain activity, defective iron–sulfur cluster assembly, and oxidative stress-driven neuronal degeneration [147,148]. In addition, *C. elegans* has provided valuable insights into mtDNA maintenance disorders through models targeting *polg-1*, the nematode ortholog of the human *POLG* gene. Deficiency or mutation of *polg-1* disrupts mitochondrial genome maintenance, resulting in mtDNA depletion or the accumulation of mtDNA mutations, impaired mitochondrial function, shortened lifespan, sterility, and widespread physiological dysfunction. These models are relevant to POLG-related primary mitochondrial disorders, which in humans commonly present with encephalopathy, ataxia, neuropathy, epilepsy, and progressive external ophthalmoplegia [149,150,151].

### 3.4. Drosophila melanogaster

The fruit fly *Drosophila melanogaster* is a powerful model organism in both fundamental and applied biomedical research [152,153,154]. Its short life cycle, high reproductive rate, and ease of genetic manipulation, supported by a fully sequenced genome, make it well suited for diverse laboratory studies. Advanced genetic tools, including the GAL4/UAS binary expression system [155], CRISPR/Cas genome editing [156], and RNA interference [157], further expand its experimental applications. Because approximately 75% of human disease-associated genes have homologs in *Drosophila*, the model has substantial translational value for investigating complex biological processes [158]. Beyond genetics, flies are also widely used in behavioral research, ranging from relatively simple traits such as locomotor activity [159] to more complex phenomena including sleep [160,161,162], aggression [163,164], and addiction [165,166,167]. These features position *Drosophila* as a model for dissecting the molecular, cellular, and behavioral mechanisms underlying human diseases. The *Drosophila* CNS contains approximately 135,000 neurons that form well-defined circuits [168]. Despite its relative simplicity compared to mammalian brains, it exhibits conserved neuronal architecture and neurotransmitter systems, including dopaminergic, serotonergic, and cholinergic networks that are also present in mammals [169,170], enhancing its translational relevance. The fruit fly has been used to study neurodevelopment, from establishing neuronal identity [171] and axon guidance [172,173], to synapse formation [174,175] and plasticity [176,177,178]. A wide range of genetic tools, such as the split-GAL4 lines, thermogenetics, and optogenetics, allows precise manipulation of specific neuronal populations [179]. In parallel, imaging approaches such as calcium- and voltage-sensitive reporters enable real-time monitoring of neuronal activity in intact, behaving flies [180,181,182]. Because of these advantages, *Drosophila* has been used to investigate synaptic transmission, molecular mechanisms of behavior, and conserved signaling pathways. Importantly, transgenic fly models mimicking human neurodegenerative disorders, including AD [183,184], PD [185], and HD [186], have enabled investigation of pathogenic mechanisms such as protein aggregation, impaired synaptic function, and neuronal loss. These studies deepen understanding of CNS biology and support preclinical testing of therapeutic strategies.

A major contribution of *Drosophila* to mitochondrial research lies in studies of mitochondrial dynamics, including fission, fusion, biogenesis, and transport [187,188]. Core regulators of these processes, such as Opa1, Mitofusin/Marf, and Drp1, are conserved in the fly, and genetic manipulation of these proteins has revealed their roles in maintaining mitochondrial morphology, distribution, and functional integrity [185,189,190]. Studies in *Drosophila* have shown that imbalances in fission and fusion cause profound defects in neuronal survival, muscle function, and lifespan, establishing direct links between mitochondrial dynamics and organismal health [191,192]. Respiratory chain deficiencies generated through targeted genetic mutations in *Drosophila* have revealed how impaired OXPHOS leads to reduced ATP production, increased ROS generation, and subsequent cellular damage [193,194,195]. These models have proven valuable for studying mitochondrial contributions to aging and for identifying antioxidant pathways that counteract oxidative stress [196]. The fly has also been used to study mitochondrial–nuclear communication, revealing how mitochondrial dysfunction triggers transcriptional reprogramming and systemic responses. For example, mitochondrial stress in *Drosophila* activates the UPRmt and alters metabolic and stress-resistance pathways, providing insight into how mitochondrial signaling integrates with organismal physiology [197,198]. *Drosophila melanogaster* is also widely used to study primary mitochondrial diseases. Many fly genes are highly conserved with their human orthologs, allowing researchers to model defects in OXPHOS, mtDNA maintenance, mitochondrial translation, and mitochondrial dynamics [199]. *Drosophila* has emerged as a valuable model for POLG-associated mitochondrial maintenance disorders. Mutations in the fly *POLG* ortholog, *tamas* (*tam*), lead to mtDNA depletion, accumulation of mitochondrial genome mutations, respiratory-chain dysfunction, locomotor impairment, and reduced lifespan, recapitulating key features of human POLG-related diseases such as Alpers-Huttenlocher syndrome and PEO [200,201,202].

Another area in which *Drosophila* has been highly informative is the study of mitochondrial dysfunction in neurodegenerative diseases and psychiatric disorders. The discovery of *Pink1* and *Parkin* pathways in *Drosophila* provided a mechanistic basis for mitophagy, the selective degradation of damaged mitochondria [185,189]. Loss-of-function mutations in *Pink1* or *Parkin* led to mitochondrial fragmentation, disrupted respiratory chain function, and dopaminergic neuronal degeneration, closely recapitulating features of PD [190,195]. These findings clarified conserved mechanisms of mitochondrial maintenance and highlighted *Drosophila* as a valuable model for mitochondrial research in neurodegeneration [197]. Similarly, *Drosophila* models of AD (expressing Aβ or tau) and HD (expressing mutant huntingtin) have demonstrated mitochondrial abnormalities, including disrupted dynamics, reduced ATP production, oxidative stress, and defective axonal transport [203,204,205,206]. These models show how mitochondrial impairment contributes to synaptic dysfunction, neuronal loss, and progressive behavioral decline. In addition to classical neurodegenerative disorders, *Drosophila* has emerged as a valuable model for psychiatric conditions, including addiction and schizophrenia [207,208,209], in which mitochondrial dysfunction is increasingly recognized as a contributing factor. Repeated exposure to ethanol or psychostimulants in flies alters mitochondrial dynamics and energy metabolism in dopaminergic circuits, mirroring findings in mammalian models. Mitochondrial dysfunction in these neurons disrupts dopamine release, reward processing, and behavioral plasticity, thereby contributing to addictive phenotypes. Moreover, *Drosophila* provides a powerful high-throughput platform to study how genetic manipulation of mitochondrial regulators (e.g., *Drp1*, *Marf*, *Pink1*) modulates drug sensitivity, tolerance, and withdrawal behaviors [210]. Table 4 summarizes key findings from *D. melanogaster* models linking CNS disorders to mitochondrial dysfunction, highlighting major genes, pathways, and phenotypes, and underscoring *Drosophila* as a powerful model for studying mitochondrial contributions to neuronal dysfunction, synaptic plasticity, and disease.

### 3.5. Danio rerio

Zebrafish (*Danio rerio*), a small tropical teleost belonging to the Cyprinidae family, has emerged as one of the most versatile vertebrate models for biomedical research [220]. Embryos develop major organs and a functional CNS within 72 h post-fertilization, while adults reach sexual maturity in approximately 12 weeks, producing hundreds of offspring weekly [221,222]. The zebrafish brain shares strong structural and functional homology with mammals, displaying conserved dopaminergic, serotonergic, cholinergic, glutamatergic, and GABAergic neurotransmitter systems [223]. At the genetic level, zebrafish share at least one ortholog with more than 70% of human genes, including numerous loci implicated in neurological and mitochondrial disorders [224]. Genetic manipulation is straightforward through microinjection and a broad range of genome-editing tools [225,226], which have generated libraries of mutant and reporter lines, facilitating detailed tracking of developmental and disease processes at subcellular resolution [227]. Additional advantages of zebrafish include high fertility, external fertilization, optical transparency during embryonic and larval stages, rapid development, a fully sequenced diploid genome, and excellent genetic and pharmacological tractability [220,226,227]. Zebrafish have emerged as a cost-effective and high-throughput vertebrate platform for modeling neurodevelopmental and neurodegenerative disorders [228]. They recapitulate key behavioral and neurochemical features observed in rodents and humans, including motor control, cognition, and anxiety-related behaviors. Moreover, many human genes linked to neurodegeneration, such as *SNCA*, *PINK1*, *Parkin*, and *LRRK2* in PD; *PSEN1* and *PSEN2* in AD; and *SOD1*, *TARDBP*, *C9orf72*, and *FUS* in ALS, have functional zebrafish orthologs [224,227,229]. Robust chemical and genetic zebrafish models of PD are well established. Exposure to neurotoxins such as 1-methyl-4-phenyl-1,2,3,6-tetrahydropyridine (MPTP) or 6-hydroxydopamine (6-OHDA) induces dopaminergic neuron loss, mimicking mammalian PD pathology [229]. Likewise, mutations in mitochondrial-associated PD genes (*pink1*, *parkin*, *DJ-1*, *LRRK2*) reproduce dopaminergic neurodegeneration and motor deficits, enabling mechanistic studies and therapeutic screening [230,231]. In ALS research, mutant *sod1* zebrafish display motor-neuron degeneration, neuromuscular-junction (NMJ) disorganization, and mitochondrial abnormalities [232], whereas *C9orf72* loss-of-function models exhibit defective synaptic vesicle release, cytoplasmic TDP-43 accumulation, and locomotor deficits [233]. AD-like phenotypes are generated through either toxin exposure or targeted genetic modifications [234]. Synthetic Aβ_42_ injection into zebrafish embryos induces cognitive impairment by day 5 post-fertilization, while okadaic acid or acrylamide trigger tau hyperphosphorylation and neuronal loss [235,236]. Insertion of the human mutant *APPswe* gene leads to Aβ accumulation, neuronal death, and enlarged perivascular spaces, recapitulating features of human AD pathology [237]. A fluorescent Tau-P301L transgenic line enables real-time visualization of tau aggregation, axonal degeneration, and cell death in live zebrafish, providing a platform for therapeutic screening [238]. Additionally, non-invasive imaging approaches, including functional MRI, micro-CT, calcium imaging, and electroencephalography, allow monitoring of neuronal activity and network dysfunction [239,240]. Chronic-stress paradigms and exposure to psychedelics, such as LSD and psilocybin, induce anxiolytic and neuroplastic responses, expanding the utility of zebrafish to psychiatric and stress-related research [241].

Because zebrafish neurons are energy-intensive and optically accessible, they are well suited for studying mitochondrial dynamics, biogenesis, and disease mechanisms in vivo [226,242]. The zebrafish and human mitochondrial genomes share approximately 65% sequence identity, identical gene order, and similar codon usage, supporting cross-species translation of results [243]. A broad range of genetic toolkits [242] and reporters, including Mitotimer, MitoFish, and fluorescent chemical probes such as MitoSOX and rhodamine dyes, enables systematic interrogation of mitochondrial physiology and real-time visualization of mitochondrial turnover, redox status, and membrane potential [244,245,246,247]. Using these approaches, zebrafish researchers have mapped ATP:ADP ratios, calcium fluxes between cytoplasm and mitochondria, and ROS dynamics under both physiological and stress conditions [248,249]. Live imaging of the zebrafish posterior lateral-line and motor neurons revealed that retrograde mitochondrial transport is essential for maintaining healthy organelle distribution, because disruption of the dynactin component actr10 arrests retrograde trafficking and leads to distal mitochondrial accumulation [250,251]. Mutant zebrafish lines targeting key mitochondrial genes replicate several human mitochondrial diseases. For example, zebrafish *polg* mutants model mitochondrial maintenance disorders by disrupting mtDNA replication and repair. TALEN-generated *polg*^−^^/^^−^ zebrafish exhibit severe mtDNA depletion, impaired mitochondrial respiration, growth abnormalities, and reduced regenerative capacity [252]. Additional *polg* models generated using antisense, ENU, and CRISPR approaches reproduce mitochondrial dysfunction and tissue-specific phenotypes, supporting studies of POLG pathogenesis and therapies [253,254]. In addition, zebrafish models of multiple acyl-CoA dehydrogenase deficiency (MADD) link mitochondrial metabolic dysfunction to neural defects, highlighting their relevance for studying CNS disorders caused by impaired bioenergetics [249]. As in humans, *Ndufb7* mutations in zebrafish cause lactic acidosis, brain malformation, and neuronal volume loss [255], whereas knockdown of *tfam*, *opa1*, *surf1*, *mfn2*, and *slc25a1* results in severe developmental abnormalities affecting the eye, heart, and brain, recapitulating features of human syndromes, including LS and MELAS (mitochondrial encephalopathy, lactic acidosis, and stroke-like episodes) [229]. Furthermore, mutations in *mfn2*, *slc25a1*, *kbp*, *kif1b*, and *actr10* mirror human Charcot–Marie–Tooth disease (CMT), producing defective mitochondrial transport and NMJ abnormalities [256,257,258]. These examples highlight zebrafish as valuable models for studying both primary mitochondrial diseases and secondary mitochondrial dysfunction in neurological disorders (Table 5).

**Figure 1:**
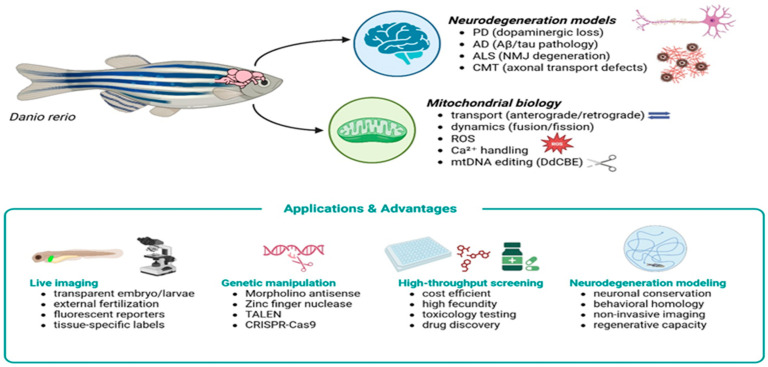
*D. rerio* as a model for CNS and mitochondria research.

Bestman et al. [259] used 2,4-dinitrophenol, an OXPHOS uncoupler, during early development to establish a zebrafish model of mitochondrial disease characterized by ATP depletion, developmental arrest, and motor-neuron and retinal abnormalities. Likewise, Pinho et al. [260] demonstrated that inhibition of the electron transport chain induces developmental and cardiovascular defects, further supporting the utility of zebrafish as a platform for studying mitochondrial pathophysiology and screening mitochondria-targeted therapeutics. Mitochondrial toxicology studies further underscore the sensitivity of zebrafish to environmental and pharmacological insults. Exposure to toxins such as chloroaniline or triphenyltin acetate [261], methylmercury [262], polycyclic aromatic hydrocarbons, arsenic, cadmium, and dioxins [263,264], as well as microcystin-LR [265], titanium dioxide and graphene oxide [266], and tramadol [267], induces mtDNA damage and oxidative stress, disrupts mitochondrial function and morphology, and provokes apoptosis. New fluorescent probes such as ZMJ214 have enabled real-time visualization of mitochondrial membrane permeabilization [268], whereas DdCBE (DddA-derived cytosine base editor)–mediated mtDNA editing in zebrafish has enabled direct modeling of mitochondrial-genome mutations affecting the CNS [269]. Furthermore, Arribat et al. [270] defined mitochondria-dependent developmental checkpoints and demonstrated that mitochondrial network distribution during zebrafish embryogenesis follows three defined spatial patterns. Wang et al. [271] showed that aging zebrafish retinas exhibit progressive declines in mtDNA integrity, fusion/fission balance, and antioxidant capacity and demonstrated therapeutic potential of resveratrol for age-related retinal degeneration. By using fluorescent dyes and genetically encoded biosensors, mitochondrial potential, Ca^2+^, ATP/ADP ratios, and ROS have been quantified in the lateral-line hair cells and afferent neurons of zebrafish [272]. In addition, Kim et al. [273] developed a transgenic zebrafish model expressing mitochondrially targeted GFP enabling visualization of organelle morphology and fragmentation following stressors such as valinomycin and carbonyl cyanide 4-(trifluoromethoxy)phenylhydrazone (FCCP). These studies establish zebrafish as a unique model for analyzing mitochondrial function, biogenesis, and disease enabling studies of disease mechanisms, therapeutic target identification, and in vivo drug screening in developmental, neurological, aging, and toxicological research (Figure 1).

## 4. Discussion

Non-mammalian model organisms have become indispensable tools for investigating mitochondrial mechanisms underlying CNS disorders because they provide complementary experimental advantages across multiple levels of biological complexity (Table 6).

Mitochondrial pathways are highly conserved across species, therefore, simpler organisms provide powerful systems for dissecting fundamental mechanisms, whereas more complex models are required to reproduce neuronal architecture, brain function, and disease-associated phenotypes. From the simplicity and high-throughput capacity of unicellular systems such as *S. cerevisiae* and *D. discoideum* to the physiological and behavioral relevance of *C. elegans*, *D. melanogaster*, and *D. rerio*, these models offer unique advantages for investigating mitochondrial function and dysfunction in CNS disorders, and their selection should be guided by the conserved biological processes each model best recapitulates (Table 7).

*Saccharomyces cerevisiae* and *Dictyostelium discoideum* represent valuable platforms for studying fundamental aspects of mitochondrial biology because of their genetic accessibility and the conservation of essential mitochondrial processes. These models have been extensively used to investigate mechanisms such as OXPHOS defects, mitochondrial protein homeostasis, mitochondrial DNA maintenance, oxidative stress responses, and mitochondrial quality-control pathways. Although they lack a nervous system, they provide important insights into cellular mechanisms underlying CNS disorders and enable the rapid identification of disease-associated pathways and potential therapeutic targets. For studies focused on mitochondrial quality control and mitophagy, particularly pathways involving PINK1, Parkin, autophagy, and lysosomal regulation, *Dictyostelium discoideum*, *Caenorhabditis elegans*, and *Drosophila melanogaster* have been highly informative. These models have contributed substantially to understanding how impaired mitochondrial clearance contributes to neuronal dysfunction, especially in PD and other neurodegenerative disorders. *Caenorhabditis elegans* provides an important transition between unicellular models and more complex animal models because it possesses a well-defined nervous system and allows direct visualization of mitochondrial behavior within neurons. Its transparent body, mapped neuronal network, genetic tractability, and short lifespan make it particularly well suited for investigating neuronal aging, mitochondrial transport, axonal degeneration, and mechanisms of neuroprotection. It has been widely employed to model PD, AD, ALS, HD, FRDA, and mitochondrial encephalopathies. *Drosophila melanogaster* is one of the most extensively used invertebrate models for mitochondrial CNS disease research because it combines genetic tools with measurable neurological phenotypes. It enables tissue-specific manipulation of mitochondrial genes and facilitates the analysis of locomotor behavior, neuronal survival, synaptic function, and neurodegeneration. Accordingly, *Drosophila* models have provided major contributions to understanding mitochondrial dysfunction in PD, AD, ALS, HD, FTD, spinocerebellar ataxias, and mitochondrial encephalomyopathies. For studies requiring greater physiological relevance to humans, *Danio rerio* offers a valuable vertebrate model. Its conserved CNS organization, rapid development, optical transparency, and compatibility with both genetic and chemical screening approaches allow real-time analysis of mitochondrial abnormalities in vivo. Zebrafish models have proven particularly useful for modeling mitochondrial encephalopathies, neurodevelopmental disorders, movement disorders, optic neuropathies, and neurodegenerative diseases. Their vertebrate nervous system provides a strong translational bridge toward mammalian studies.

Although no single non-mammalian model organism can fully recapitulate the complexity of human mitochondrial disorders affecting the CNS, their complementary use has enabled major advances in understanding of conserved mitochondrial pathways, including mitophagy, mitochondrial quality control, bioenergetic regulation, signaling networks, and neurodegenerative mechanisms. Features such as genetic tractability, rapid life cycles, live imaging capabilities, and suitability for high-throughput screening have accelerated both CNS disease modeling and therapeutic discovery. Furthermore, non-mammalian animal models are consistent with ethical frameworks and the principles of the 3Rs in animal experimentation, serving as effective alternatives to traditional mammalian models, thereby minimizing ethical concerns related to the use of higher vertebrates. Although each system has limitations arising from evolutionary distance, simplified nervous systems, or reduced translational complexity, their use in complementary and sequential research provides opportunities for mechanistic studies and preclinical therapeutic discovery. Collectively, these models continue to link fundamental mitochondrial biology with translational neuroscience, advancing understanding of CNS disorders and facilitating the development of novel therapeutic approaches.

## 5. Conclusions

Non-mammalian model organisms have become indispensable tools for advancing our understanding of mitochondrial dysfunction in CNS disorders by offering diverse and complementary experimental advantages. Models ranging from the unicellular systems such as *Saccharomyces cerevisiae* and *Dictyostelium discoideum* to the multicellular organisms including *Caenorhabditis elegans*, *Drosophila melanogaster*, and the vertebrate *Danio rerio* provide experimental platforms spanning different levels of biological complexity. Their use has generated major discoveries in conserved mitochondrial pathways, including mitophagy, mitochondrial quality control, respiratory function, bioenergetic signaling, and neurodegenerative mechanisms that are central to human CNS pathology. Their key strengths, including genetic tractability, rapid life cycles, optical transparency for live imaging, scalability, and compatibility with high-throughput screening, have enabled efficient disease modeling and accelerated therapeutic discovery. On the other hand, each model also possesses limitations arising from simplified physiology, evolutionary distance from humans, and incomplete representation of complex CNS organization. Consequently, findings from these systems often require validation in mammalian models. Nevertheless, integrating them with complementary research offers a powerful approach for linking fundamental mitochondrial biology with translational neuroscience. Technological advances, including genome editing, single-cell and spatial omics technologies, and advanced in vivo imaging, will further expand the utility of these models, accelerating mechanistic discoveries and the development of novel therapeutic strategies for mitochondrial dysfunction in CNS disorders.

## Figures and Tables

**Table 1:** Selected *S. cerevisiae* models of CNS disorders with mitochondrial malfunction.

Model for	Model Description	Key Findings	References
HD	Expression of huntingtin fragments containing flanking polyQ regions	Defective mitochondrial transport, dynamic imbalances, modulated conversion of benign to toxic protein aggregates	[21,24]
PD	Inducible expression of α-synuclein fused to GFP (galactose-regulated), expression of mitochondrial Hsp70 variants (P509S, R126W)	Impaired ER-to-Golgi protein trafficking leads to mitochondrial stress; enhanced interaction and activation of the mutated mtHsp70 variant with mitochondrial J-proteins, protein instability, increased intracellular protein aggregation	[26,32]
ALS	Expression of human mutant SOD1 variants replacing the native yeast ortholog (sod1Δ)	Disrupted protein stability and conformation due to altered acetylation/amino acid modifications, possible impaired antioxidant protection in the mitochondrial intermembrane space where SOD1 also localizes in addition to the cytoplasm	[25,33,34]
LS	Expression of novel single-point OPA1 mutations or splice variants in the ATP5PO gene	Validation of disease-associated mitochondrial pathogenicity and structural defects	[35,36]
Ataxia, NARP, and MILS	Models harboring point mutations in the ATP6 gene (MT-ATP6 ortholog)	Severe impairment of ATP synthase function leading to downstream reduction in Complex IV levels	[37,38,39,40]

ALS—Amyotrophic lateral sclerosis; HD—Huntington’s disease; LS—Leigh syndrome; MILS—maternally inherited Leigh syndrome; NARP—neuropathy, ataxia, and retinitis pigmentosa; PD—Parkinson’s disease.

**Table 2:** *D. discoideum* as a model for primary mitochondrial dysfunction and PD.

Model for	Model Description	Key Findings	References
Mitochondrial dysfunction induced by incorrect folding and assembly of mitochondrial proteins	Reduced expression of chaperonin 60 (Cpn60)	KO is lethal, the KD phenotype is consistent with respiratory dysfunction (severe defects in slug phototaxis and thermotaxis, growth defects not due to impaired phagocytosis or macropinocytosis, impaired development), chronic activation of AMPK is responsible for the phenotype	[65,66,83]
Mitochondrial dysfunction induced by CI deficiency	Deficiency of MidA (Ndufaf7)	The KO phenotype is not fully consistent with mitochondrial dysfunction (severely reduced growth on bacterial lawns but only moderately reduced axenic growth, reduced phagocytosis and macropinocytosis, developmental defects with reduced viability of spores, strong defect in slug phototaxis and thermotaxis), reduced ATP levels, a 50% reduction in CI activity, partially dependent on AMPK activation	[68,84,85]
PD	Reduced expression and overexpression of wild type and protease-dead mutant of HtrA2	KD and OE of the protease-dead mutant have the same phenotype, which is not fully consistent with mitochondrial dysfunction (reduced growth on bacterial lawns, mildly reduced axenic growth, aberrant fruiting body morphology, no defect in slug phototaxis), no respiratory defects, OE of wild type protein is lethal due to high protease activity	[79,86,87]
PD	Deficiency and increased kinase activity of Roco4	KO and increased kinase activity (conserved PD mutation): severe developmental defects, strong defect in slug phototaxis associated with mitochondrial dysfunction; KO: increased basal respiration, ATP synthesis and CI activity; increased kinase activity: reduced basal and maximal respiration	[75,78,88]

CI—Complex I; KD—knockdown; KO—knockout; OE—overexpression; PD—Parkinson’s disease.

**Table 3:** Selected *C. elegans* models of CNS disorders and mitochondrial phenotypes.

Model for	Model Description	Key Findings	References
PD	α-synuclein expression; pink-1 or pdr-1 mutants	Fragmented mitochondria, impaired mitophagy, respiration defects, excessive ROS production, dopaminergic neuron loss	[112,139]
AD	Neuronal Aβ or tau expression	ATP depletion, increased ROS, mitochondrial fragmentation, disrupted calcium homeostasis, synaptic failure, neuronal dysfunction, phenotypes mitigated by UPRmt activation and HSP-6 and HSP-60 upregulation	[140,141,142]
HD	PolyQ expansion models	Defective mitochondrial transport, altered fission–fusion balance, impaired respiratory capacity, elevated ROS levels in GABAergic and cholinergic neurons, behavioral and locomotor impairments	[143,144,145]
ALS	Mutant SOD1 or TDP-43 expression	Impaired mitochondrial transport along axons, accumulation of oxidatively damaged mitochondria, oxidative stress, defective mitophagy, impaired axonal transport, motor neuron degeneration	[125,146]
LS	Reduced mma-1 (LRPPRC) function	Mitochondrial hyperfusion, decreased activity of complex IV of the electron transport chain	[147]
FRDA	Frataxin homolog (frh-1) knockdown or deletion mutants; RNAi-mediated frataxin suppression	Reduced mitochondrial respiration, mitochondrial iron accumulation, defective Fe–S cluster biogenesis, elevated ROS levels, impaired ATP production, neuronal degeneration, locomotor dysfunction	[148]
POLG-related mtDNA maintenance disorders	polg-1 deficiency or mutant polg-1 models	mtDNA depletion or accumulation of mtDNA mutations, impaired mitochondrial genome maintenance, reduced mitochondrial function, shortened lifespan, sterility and organismal dysfunction	[149,150,151]

ALS—Amyotrophic lateral sclerosis; FRDA—Friedreich’s ataxia; HD—Huntington’s disease; LS—Leigh syndrome; mtDNA—mitochondrial DNA; PD—Parkinson’s disease; POLG—mitochondrial DNA polymerase γ catalytic subunit.

**Table 4:** Key studies in *D. melanogaster* on mitochondrial dysfunction in CNS disorders.

Model for	Model Description	Key Findings	References
PD	*Pink1* mutants	Severe mitochondrial fragmentation and swelling, disrupted cristae, impaired mitochondrial function, defective mitochondrial quality control and mitophagy, dopaminergic neuron degeneration, locomotor defects	[185]
PD	*Pink1/Parkin* pathway	PINK1-dependent recruitment of Parkin to damaged mitochondria activates mitophagy, conserved mitochondrial quality control pathway in vivo	[190]
PD	*Pink1*/*Parkin* with ref(2)P mutants	Defective mitophagy due to impaired autophagic clearance of damaged mitochondria, persistent mitochondrial dysfunction	[211]
PD/Aging	Aging fly tissues with *Pink1/Parkin* deficiency	Age-dependent decline in mitophagy, accumulation of damaged mitochondria, impaired mitochondrial turnover, progressive tissue and neuronal dysfunction	[212]
AD	Neuronal Aβ42 expression	Impaired mitochondrial transport, depletion of mitochondria from axons and dendrites and accumulation in neuronal soma, preceding neuronal dysfunction	[213]
AD	Aβ42 expression	Disrupted mitochondrial homeostasis associated with impaired autophagy/mitophagy, accumulation of autophagic vesicles, age-dependent neuronal dysfunction	[214]
HD	Mutant huntingtin expression	Impaired mitochondrial axonal transport, disrupted mitochondrial dynamics and fission–fusion balance, bioenergetic defects, synaptic dysfunction	[215]
HD	*Drosophila* HD models	Genetic restoration of mitochondrial function, including Parkin activation rescues mitochondrial defects and improves neuronal and muscle phenotypes	[216]
ALS/FTD	TDP-43 overexpression or mutations	Altered mitochondrial dynamics and distribution, impaired mitochondrial function, mitochondrial contribution to neurodegeneration and neuronal loss	[217]
ALS	*Sod1* mutant	Early compartment-specific mitochondrial dysfunction preceding overt symptoms and contributing to motor neuron vulnerability	[218]
Addiction	Single-dose ethanol exposure	Altered mitochondrial trafficking required for reward memory formation, disruption of mitochondrial transport abolishes ethanol-induced reward behavior	[219]

AD—Alzheimer’s disease; ALS—Amyotrophic lateral sclerosis; FTD—Frontotemporal Dementia; PD—Parkinson’s disease.

**Table 5:** Selected *D. rerio* models for mitochondrial and CNS-related disorders.

Model for	Model Description	Key Mitochondrial Findings	References
PD	Neurotoxin-based models using MPTP or 6-OHDA; genetic models involving *pink1*, *Parkin*, *DJ-1*, and *LRRK2*-associated pathways	Vulnerability to oxidative stress, upregulation of genes associated with OXPHOS, dopaminergic neuron loss, altered motor behavior, mitochondrial-associated PD phenotypes	[229,230,231]
ALS	Mutant *sod1* zebrafish; *c9orf72* loss-of-function model	Motor-neuron degeneration, NMJ disruption, mitochondrial vacuolation, impaired synaptic vesicle release, TDP-43 mislocalization, locomotor impairment	[232,233]
AD	Aβ42 injection, okadaic acid exposure, human mutant *APP* (*APPswe*) transgenic zebrafish, *Tau-P301L* transgenic line	Cognitive impairment, increased tau phosphorylation and aggregation, Aβ accumulation, neuronal death, enlarged perivascular spaces, axonal degeneration	[234,235,236,237,238]
Complex I dysfunction	Human *NDUFB7*-related mitochondrial disease modeled using ndufb7-deficient zebrafish	Lactic acidosis, brain malformation, reduced neuronal volume, mitochondrial dysfunction	[255]
LS MELAS	Knockdown or mutation of mitochondrial genes including *tfam*, *opa1*, *surf1*, *mfn2*, and *slc25a1*	ROS overproduction, mitochondrial dysfunction, severe developmental abnormalities affecting the eye, heart, and brain	[229]
MADD	Zebrafish models compared with human MADD mechanisms	Metabolic and neural defects associated with impaired mitochondrial fatty-acid oxidation and mitochondrial dysfunction	[249]
CMT	*mfn2*, *slc25a1*, *kbp*, *kif1b*, and *actr10* models	Defective mitochondrial transport, reduced motile mitochondria, accumulation of mitochondria, abnormal motor neurons, impaired neuromuscular transmission, NMJ pathology	[229,251,256,257,258]
POLG-related mitochondrial disease	polg^−/−^ TALEN, polg antisense/CRISPR, polg2 mutant line (polg2ia304) models	mtDNA depletion, altered mitochondrial network and dynamics, reduced mitochondrial respiration, impaired growth and regeneration	[252,253,254]

AD—Alzheimer’s disease; ALS—Amyotrophic lateral sclerosis; CMT—Charcot–Marie–Tooth disease; LS—Leigh syndrome; MADD—multiple acyl-CoA dehydrogenase deficiency; MELAS—mitochondrial encephalopathy, lactic acidosis, and stroke-like episodes; MPTP-1—methyl-4-phenyl-1,2,3,6-tetrahydropyridine; mtDNA—mitochondrial DNA; NMJ—neuromuscular junction; PD—Parkinson’s disease; POLG—mitochondrial DNA polymerase γ catalytic subunit; 6-OHDA—6-hydroxydopamine.

**Table 6:** Key features, advantages, limitations, translational relevance, and applications of non-mammalian models in mitochondrial and CNS research.

Model Organism	Key Characteristics	Advantages	Limitations	CNS Disease Investigated	Translational Relevance	Research Applications
*Saccharomyces* *cerevisiae*	Single-celled eukaryote; highly conserved mitochondrial genome and pathways; no nervous system	Rapid growth; low cost; powerful genetic manipulation; high-throughput screening; well-characterized mitochondrial biochemistry, allows study of mitochondrial defects (loss of OXPHOS) incompatible with higher organisms	No neurons; no nervous system; cannot model neuronal circuits, behavior or neuroinflammation; lack of cell–cell interactions; limited disease phenotyping	PD (α-synuclein toxicity, PINK1/Parkin pathways); AD (Aβ and tau toxicity studies); HD (polyQ toxicity); ALS (TDP-43 and FUS toxicity); mitochondrial encephalopathies (OXPHOS defects); POLG-related mitochondrial disease	Identifies conserved cellular mechanisms underlying neurodegeneration, but requires validation in neuronal models	Mitochondrial biogenesis; OXPHOS; mtDNA maintenance; protein import and folding; mitophagy; oxidative stress; pathogenic variant testing; drug-target identification
*Dictyostelium* *discoideum*	Social amoeba; unicellular organism with multicellular developmental stages; conserved mitochondrial quality-control pathways	Intermediate complexity between yeast and animals; Easy genetic manipulation; suitable for studying autophagy, mitophagy, and stress responses	No nervous system; limited disease-specific phenotypes	PD (PINK1, Parkin, LRRK2 studies); HD (mutant huntingtin toxicity); rare mitochondrial disorders affecting energy metabolism	Useful for dissecting evolutionarily conserved mitochondrial and lysosomal pathways relevant to neurodegeneration	Mitophagy; mitochondrial dynamics; autophagy; oxidative stress; lysosomal–mitochondrial interactions
*Caenorhabditis* *elegans*	Multicellular nematode; 302 neurons; transparent body; conserved mitochondrial pathways and aging mechanisms	Fully mapped nervous system; live imaging; RNA interference; short lifespan enables aging studies; easy generation of transgenic lines; robust neurodegeneration models	Simple nervous system lacking mammalian brain complexity and regions and vertebrate immune interactions	PD (PINK1, Parkin, DJ-1); AD; ALS; HD; FRDA; LS; CMT; mitochondrial complex I deficiency; age-related neurodegeneration; POLG-related mitochondrial disease	Model for conserved pathways linking mitochondrial dysfunction to neuronal degeneration and behavior	Aging; mitochondrial transport; proteostasis, neurodegeneration; behavioral phenotyping; drug screening; stress-response
*Drosophila* *melanogaster*	Multicellular animal, invertebrate; complex brain structures; conserved mitochondrial genes and neurotransmitter systems; sophisticated genetic tools	Cell-specific gene manipulation; behavioral phenotyping; rapid generation time; models neuronal degeneration and locomotor defects; high-throughput genetic screens	Lacks mammalian brain complexity and architecture; no adaptive immune system; differences in metabolism and drug processing	PD (PINK1, Parkin, DJ-1, LRRK2); AD; ALS; HD; FTD; LS; mitochondrial encephalomyopathies; optic neuropathies; POLG-related mitochondrial disease	Highly informative for mechanisms linking mitochondrial dysfunction to neuronal degeneration and behavior	Mitochondrial dynamics; neuronal energy metabolism; synaptic dysfunction; genetic modifier screens; neurodegeneration mechanisms; behavioral phenotyping; therapeutic testing
*Danio* *rerio*	Vertebrate with conserved CNS organization, transparent embryos, and human-like mitochondrial genetics	Vertebrate nervous system; rapid development; live imaging of neurons and mitochondria, CRISPR editing, high-throughput drug screening	Lower CNS complexity than mammals; some physiological differences	PD; AD; ALS; HD; LS; MELAS; mitochondrial complex I and IV deficiencies; optic neuropathies; neurodevelopmental disorders; POLG-related mitochondrial disease	Strong bridge between invertebrate and mammalian models; allows visualization of CNS pathology in vivo; models disease phenotypes	Mitochondrial encephalopathies, neurodevelopmental disorders, neuronal survival, mitochondrial trafficking, drug discovery; precision genetic models

AD—Alzheimer’s disease; ALS—Amyotrophic lateral sclerosis; FRDA-Friedreich’s ataxia; FTD—Frontotemporal Dementia; LS—Leigh syndrome; MELAS—mitochondrial encephalopathy, lactic acidosis, and stroke-like episodes; mtDNA—mitochondrial DNA; PD—Parkinson’s disease; POLG—DNA polymerase γ catalytic subunit.

**Table 7:** Conservation of key biological processes across non-mammalian model organisms.

Biological Process	*Saccharomyces cerevisiae*	*Dictyostelium* *discoideum*	*Caenorhabditis elegans*	*Drosophila* *melanogaster*	*Danio* *rerio*
OXPHOS	High—Conserved respiratory chain; lacks Complex I [274].	Moderate–High—Conserved mitochondrial respiratory pathways [42].	High—Conserved mitochondrial respiration [275].	High—Conserved mitochondrial bioenergetics [193].	High—Models mitochondrial respiratory defects [276].
mtDNA maintenance	High—Conserved genome inheritance/stability [277].	Moderate—Genome organization conserved; mechanisms less defined [42].	High—Developmental regulation of mtDNA [278].	High—Conserved mtDNA maintenance [279].	High—Conserved mtDNA metabolism [280].
Mitochondrial dynamics	High—Dnm1p fission/Fzo1p fusion [281].	Moderate–High—Conserved fission/fusion pathways [282].	High—DRP-1-dependent fission [283].	High—PINK1/Parkin regulates morphology [284].	High—Conserved mitochondrial fusion/fission pathways [285].
Mitophagy/ mitochondrial quality control	Moderate—Atg32-dependent mitophagy; no PINK1/Parkin [286].	Low–Moderate—Autophagy conserved; selective mitophagy less defined [55].	High—PINK-1/PDR-1 quality control [135].	High—PINK1/Parkin pathway conserved [284].	High—Conserved PINK1-dependent mitochondrial quality control [287].
ROS signaling/oxidative stress	High—Conserved stress responses [288].	Moderate—Conserved ROS responses and antioxidant mechanisms [289].	High—Oxidative stress resistance pathways [290].	High—ROS regulates stress responses [291].	High—ROS responses and antioxidant pathways conserved [292].
Mitochondrial transport	Low—Mitochondrial inheritance during budding [293].	Moderate—Microtubule-dependent mitochondrial motility [282].	High—Neuronal mitochondrial transport [294].	High—Milton-dependent transport [295].	High—Axonal mitochondrial transport in vivo [296].
Synaptic mitochondrial function	N/A—No nervous system.	N/A—No nervous system.	Moderate—Neuronal mitochondrial function [297].	High—Synaptic mitochondria regulate neurotransmission [298].	Moderate–High—Mitochondrial function supports neuronal survival [287].
Neuroimmune/immune responses	N/A—No nervous or immune system.	Low–Moderate—Phagocytosis and innate immunity-like responses [299].	Moderate—Innate immunity conserved [300].	Moderate—Toll-mediated immunity conserved [301].	High—Innate and adaptive immunity conserved [302].

High—core conserved mitochondrial processes; Moderate—conserved but lineage-specific differences; Low—limited homology or major functional divergence; N/A—not applicable, absent biological system; mtDNA—mitochondrial DNA; OXPHOS—oxidative phosphorylation; ROS—reactive oxygen species.

## Data Availability

No new data were created or analyzed in this study. Data sharing is not applicable.

## Abbreviations

The following abbreviations are used in this manuscript:

3Rs-	Replacement, reduction, and refinement
6-OHDA	6-hydroxydopamine
AD	Alzheimer’s disease
ADP	Adenosine diphosphate
ALS	Amyotrophic lateral sclerosis
AMPK	AMP-activated protein kinase
ATP	Adenosine triphosphate
Aβ	Amyloid-β
CI	Complex I
CMT	Charcot–Marie–Tooth disease
CNS	Central nervous system
CRISPR	Clustered regularly interspaced short palindromic repeats
DA	Dopamine
DdCBE	DddA-derived cytosine base editor
DNA	Deoxyribonucleic acid
ER	Endoplasmic reticulum
FCCP	Carbonyl cyanide 4-(trifluoromethoxy)phenylhydrazone
FRDA	Friedreich’s ataxia
FTD	Frontotemporal dementia
GFP	Green fluorescent protein
HD	Huntington’s disease
IIS	Insulin/IGF-1 signaling
KD	Knockdown
KO	Knockout
LHON-	Leber hereditary optic neuropathy
LS	Leigh syndrome
LSD	Lysergic acid diethylamide
MADD	Multiple acyl-CoA dehydrogenase deficiency
MELAS	Mitochondrial encephalopathy, lactic acidosis, and stroke-like episodes
Micro-CT	Micro-computed tomography
MILS	Maternally inherited LS
MPP^+^	1-methyl-4-phenylpyridinium
MPTP	1-methyl-4-phenyl-1,2,3,6-tetrahydropyridine
MRI	Magnetic resonance imaging
mtDNA	Mitochondrial DNA
N/A	Not applicable
NARP	Neuropathy, ataxia, and retinitis pigmentosa syndrome
nDNA	Nuclear DNA
NCL	Neuronal ceroid lipofuscinosis
NIH	National institutes of health
NMJ	Neuromuscular junction
OE	Overexpression
OXPHOS	Oxidative phosphorylation
PD	Parkinson’s disease
PEO	Progressive external ophthalmoplegia
POLG	DNA polymerase γ catalytic subunit
PolyQ	Polyglutamine
RNA	Ribonucleic acid
ROS	Reactive oxygen species
TALEN	Transcription activator-like effector nuclease
UPRmt	Mitochondrial unfolded protein response
